# A new genus and species of termites (Isoptera, Termitidae, Nasutitermitinae) from the rainforest of northern Peru

**DOI:** 10.3897/zookeys.159.2311

**Published:** 2011-12-23

**Authors:** Carolina Cuezzo, David A. Nickle

**Affiliations:** 1 CONICET- Instituto Superior de Entomología “Dr. A. Willink”, Facultad de Ciencias Naturales e Instituto Miguel Lillo, UNT, Miguel Lillo 205, T4000JFE, San Miguel de Tucumán, Argentina; 2 Systematic Entomology Laboratory, PSI, Agricultural Research Service, U.S. Department of Agriculture Beltsville Agricultural Research Center, Building 005, Rm. 137, Beltsville, MD 20705-2350, USA

**Keywords:** Taxonomy, *Subulitermes*-group, enteric valve configuration, gut coiling, Peruvian rainforest

## Abstract

A new genus and species of nasutitermitine termites are described and illustrated, based on soldier and worker characters. *Sinqasapatermes* **gen. n.**, can be distinguished from all other nasutitermitine genera by its singular worker gut coiling and enteric valve characters: distal margin of the enteric valve not everted into the paunch but bending towards the ileum, that is, directed against the flow of food; enteric valve armature with one ring of six equal subtriangularly-shaped ridges, each ridge with short spines on the entire surface; enteric valve armature situatedonexternal face of cone, facing the internal ileum wall; enteric valve seating tri-lobed and separated from remaining portionof the paunch; paunch subdivided. *Sinqasapatermes sachae* **sp. n.**, was collected on a tree in a very narrow flattened tunnel that was well concealed beneath lichens in a northern Peru rainforest (Arcadia, Loreto Province).

## Introduction

The subfamily Nasutitermitinae is a monophyletic group, currently worldwide represented by 81 living genera and 575 living species (Constantino 2011). It is the second largest subfamily in terms of generic and specific diversity within Termitidae. New World representatives of the subfamily Nasutitermitinae account for approximately a third of the total generic and specific diversity, being the dominant group of termites in South America.

Although Nasutitermitinae was never formally subdivided into minor taxonomic hierarchies, several authors (Kovoor 1969; Noirot 2001) have recognized three groups of genera within this subfamily based on gut morphology: the *Nasutitermes*-group, the *Subulitermes*-group, and the *Syntermes*-group. Recently, Engel and Krishna (2004) raised the *Syntermes*-group to the subfamilial level, named Syntermitinae.

Currently, there are 21 genera described as part of the *Subulitermes*-group, with 11 genera occurring in the Neotropical region (Holmgren 1910; Emerson 1925; Snyder 1926; Araujo 1970; Fontes 1979, 1982; Constantino 1990, 1991; Cancello and Noirot 2003), two genera in the Australian region (Emerson 1960a; Miller 1984), and eight genera in the Ethiopian region (Emerson 1960b; Coaton 1971; Sands 1965, 1998).

In this study, an unusual termite sample collected from the rainforest of northern Peru is described and assigned to a new neotropical nasutitermitine genus and species, *Sinqasapatermes sachae* gen. n. and sp. n. The systematic position of the new genus is determined according to an evaluation of the internal morphology. Such characters provide enough evidence to place *Sinqasapatermes* gen. n., in the *Subulitermes*-group and also to distinguish it substantially from other known nasutitermitine genera within this group. The phylogenetic relationships among members of the *Subulitermes*-group remain unresolved (Inward et al. 2007), and it is well known that the taxonomic validity of some neotropical genera is questionable (Roisin 1995; Cancello and Noirot 2003). We do not intend to resolve the systematic relationships within the *Subulitermes*-group, but to contribute to a better understanding of this important lineage by describing this singular new genus and species.

## Material and methods

The sample was subdivided and deposited in the Isoptera Collection of the Museo de Historia Natural, Universidad Nacional Mayor de San Marcos, Lima, Peru (MUSM), the National Museum of Natural History, Washington, DC, USA (USNM) and the Museu de Zoologia da Universidade de Sao Paulo, Brazil (MZUSP).

Morphometric characters used in this study and their correspondence with Roonwall’s system (Roonwall 1970) are indicated in parentheses as follows: LH, length of head with nasus (12); LHp, length of head to apex of postclypeus (14, but in profile); WH, width of head (17); HH, height of head excluding postmentum (21); WP, width of pronotum (68); and LT, length of hind tibia (85). Left mandible index was not calculated; instead the distance between the apical tooth to M1+2 was measured.

Description of the digestive tube morphology follows Noirot (2001), and those of the Malpighian tubules follow Sands (1998). Worker mandible descriptions are based on terminology used by Fontes (1987). The terms “bristles” and “hairs” are defined as follows: “long bristles”, longer than length of first antennal article; “short hairs”, visible at about 12× magnification; “microscopic hairs”, visible at 50× magnification.

Drawings were made with a camera lucida attached to a stereomicroscope.

## Taxonomic treatment

### Sinqasapatermes gen. n.

urn:lsid:zoobank.org:act:15D04831-D8C0-4059-BC6D-75E77AAA7C46

http://species-id.net/wiki/Sinqasapatermes

#### Type-species.

*Sinqasapatermes sachae* sp. n.

#### Diagnosis.

The new genus is distinguished from other nasutitermitine genera by the unique combination of the following characteristics from the worker digestive tube: distal margin of the enteric valve (P2) not everted into the paunch (P3) but bending towards the ileum (P1), that is, directed against the flow of food; armed with one ring of six equal subtriangularly-shaped ridges, each ridge with short spines on entire surface (Fig. 14); enteric valve armature situatedonexternal face of cone, facing P1 internal wall (Fig. 15); enteric valve seating tri-lobed and separated from remaining portionof P3; P3 subdivided.

#### Description.

*Imago*. Unknown.

*Soldier*. Monomorphic. In dorsal view, head capsule with a constriction behind base of antenna. Maximum width behind constriction, at middle of posterior lobe. In profile, dorsal margin of head capsule nearly straight to base of nasus; weakly depressed at base of nasus. Nasus narrow, conical in dorsal view; about same length as head capsule; slanted slightly upward in profile. Mandibles vestigial, without points. Postclypeus moderately arcuate, in profile. Labrum wider than long, with rounded antero-lateral corners. Antenna with 11 articles. Pronotum shallowly saddle-shaped, anterior margin rounded, not emarginate. Tibial spurs 2:2:2.

*Worker*. Head capsule trapezoidal in dorsal view; maximum width of head capsule at base of mandibles. Fontanelle area depressed, situated at posterior third of head capsule.Postclypeus short and moderately inflated; median line weakly defined. Antenna with 12 articles. Pronotum shallowly saddle-shaped, anterior margin rounded, not emarginate. Tibial spurs 2:2:2.

Mandibles. Left mandible (Fig. 5) with apical tooth slightly as prominent as or slightly more prominent than M1+2, posterior margin of apical tooth weakly concave or truncate; right or acute angle between posterior margin of apical tooth and anterior margin of M1+2; posterior margin of M1+2 straight or slightly sinuous; third marginal tooth reduced to a vestigial node; molar tooth visible at V-shaped gap but apex hidden beneath molar prominence; molar prominence with very weakly-defined ridges. Right mandible (Figs 6–7) with apical tooth slightly more prominent than first marginal tooth, second marginal tooth not visible or vestigial (as a minute prominence); molar plate concave with very weakly defined ridges; basal notch well defined.

Digestive tube (Figs 8–15). Coiling gut *in situ* forming a short, broad mass. Crop (C) voluminous, partially visible in dorsal view; positioned to left halfof abdomen. Gizzard (G) with a strong musculature; well separated from crop; partially visible in ventral view. Mesenteron (M) passing through right side but not reaching medial line in ventral view. Mesenteric-proctodeal junction circular (mixed segment absent); visible in right lateral view. Malpighian tubules (TM) slightly dilated to form an ampulla at base, arranged in adjacent pairs with a common base at mesenteric-proctodeal junction on inner face. Ileum or first proctodeal segment (P1) tubular; shorter than mesenteron length. Enteric valve (P2) lying beneath P1, conical with distal margin not everted into P3 but bending towards P1, i.e., directed against the flow of food (Fig. 15); armed with one ring of six equal subtriangularly-shaped ridges, each ridge with short spines on entire surface (Fig. 14); enteric valve armature situatedonexternal face of cone, facing P1 internal wall (Fig. 15). P2 in same axis aspaunch (P3). Enteric valve seating, tri-lobed and separated from remaining portionof P3 by a constriction; another subdivision visible at P3 before protruding through mesenteric ring; distal part of P3 very prominent in dorsal view and joining colon (P4) on left side; isthmus conspicuous. Dorsal torsion well developed. P4 “U-turn” dilated. Distal colon tubular.

#### Etymology.

From Quechua, indigenous South American language, *sinqa* = nose and *sapa* = big, and Latin *termes* = termite, meaning termite with a big nose.

#### Comparisons.

Among soldiers of neotropical genera, the long,narrow nasus and reduced number or absence of bristles on head capsule and thoracic nota are shared characters among species of *Cyranotermes* Araujo, *Anhangatermes* Constantino and *Sinqasapatermes*, but *Cyranotermes* and *Anhangatermes* species have antenna with 13 articles and a rounded head capsule. Soldiers of the latter two genera are also significantly larger than those of *Sinqasapatermes*. *Agnathotermes* Snyder and *Paraconvexitermes* Cancello & Noirot also have a conical nasus and antenna with 11 articles, but they differ from *Sinqasapatermes* by their chaetotaxy and head shape of the soldier.Soldiers of species of *Angularitermes* Emerson, *Araujotermes* Fontes, *Atlantitermes* Fontes, *Coatitermes* Fontes, *Convexitermes* Holmgren, *Ereymatermes* Constantino and *Subulitermes* Holmgren are genera that can be separated from *Sinqasapatermes* on the basis of different chaetotaxy arrangement and head capsule shape.

Among workers of neotropical genera, the third marginal tooth of left mandible and second marginal tooth of right mandible absent or vestigial are shared characters among species of *Cyranotermes* and *Anhangatermes*, however, molar area differs in *Sinqasapatermes* by having no ridges and being more concave. Reduced ridges on molar areas are found in *Araujotermes*, *Atlantitermes*, *Coatitermes*, *Convexitermes*, *Ereymatermes*, *Paraconvexitermes*, and *Subulitermes*, but none of these genera have lost the marginal teeth. The worker gut coiling and enteric valve features of *Sinqasapatermes* do not match any member of the *Subulitermes*-group (Kovoor 1969; Fontes 1987; Constantino 1990, 1991; Roisin 1995; Sands 1998; Cancello and Noirot 2003).

### Sinqasapatermes sachae sp. n.

urn:lsid:zoobank.org:act:86BBA560-790D-42D8-B3EF-FFB3864F534C

http://species-id.net/wiki/

Figs 1 15

#### Type material.

Holotype soldier, in alcohol, separate in a microvial. Original typewritten label: “PERU, Loreto, Arcadia, 0°59.37'S, 75°18.55'W, 150m, 31 Oct-10 Nov. 1993 leg., D.A. Nickle & J. Lewis; Castana 227; E66". Holotype will be deposited at the Museo de Historia Natural, Universidad Nacional Mayor de San Marcos, Lima, Peru (MUSM). Paratypes: 12 soldiers and 14 workers with same data as holotype. Paratypes will be deposited as follows: five soldiers and six workers at the MUSM, five soldiers and six workers as part of lot no. USNM 10601 at the USNM; two soldiers and two workers as part of lot no. MZUSP 13095 at the MZUSP.

#### Type locality.

PERU, Loreto Province: Arcadia, 0°59.37'S, 75°18.55'W, 190m. Type material was collected on a tree in a very narrow flattened tunnel that was well concealed beneath green/white lichens at the rainforests of northern Peru. The tunnel was not obvious.

#### Diagnosis.

Monotypic genus – see generic diagnosis.

#### Description.

*Imago*. Unknown.

**Figures 1–7.**
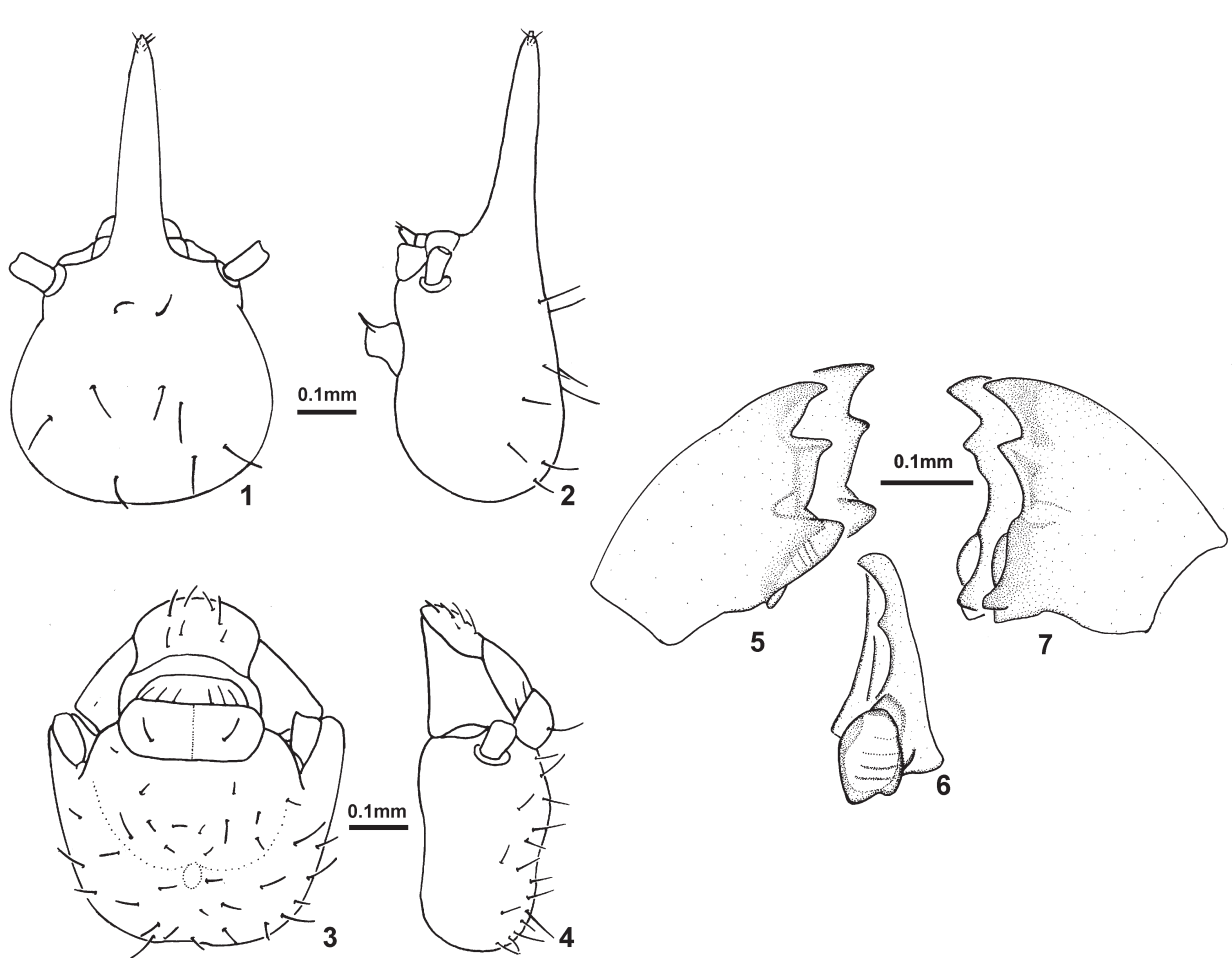
*Sinqasapatermes sachae* **1** soldier head in dorsal view **2** soldier head in profile **3** worker head in dorsal view **4** worker head in profile **5** worker left mandible in dorsal view **6** worker right mandible, showing molar plate in frontal view **7** worker right mandible in dorsal view.

*Soldier* (Figs 1–2). Head capsule with scattered long bristles and a few short hairs. Nasus with four shorter bristles and a few microscopic hairs near apex. Labrum with four short hairs. Postclypeus and postmentum glabrous. Thoracic nota and abdominal tergite I glabrous. Tergite II with a few long bristles at posterior margin; tergites III–X with several long bristles at posterior margin; IX–X tergites with bristles and hairs distributed on entire surface. Sternites with more bristles than tergites. Legs with two long bristles, first proximal and second at middle on external surface of all tibiae; short hairs on internal surface of all tibiae. Head capsule yellow; nasus reddish; antenna yellow with articles III–IV reddish; thoracic nota pale yellow; digestive tube visible through abdominal sclerites. Measurements (mm) of six soldiers are given as ranges, followed by holotype values in parentheses: LH, 0.85–0.90 (0.88); LHp, 0.46–0.49 (0.48); WH, 0.43–0.48 (0.48); HH, 0.30–0.32 (0.32); WP, 0.25–0.27 (0.26); LT, 0.48–0.51 (0.49). Ratios: LH/WH, 1.83–1.98 (1.83); LH/LT, 1.74–1.80; LHp/LT, 0.94–0.98 (0.98).

**Figures 8–15.**
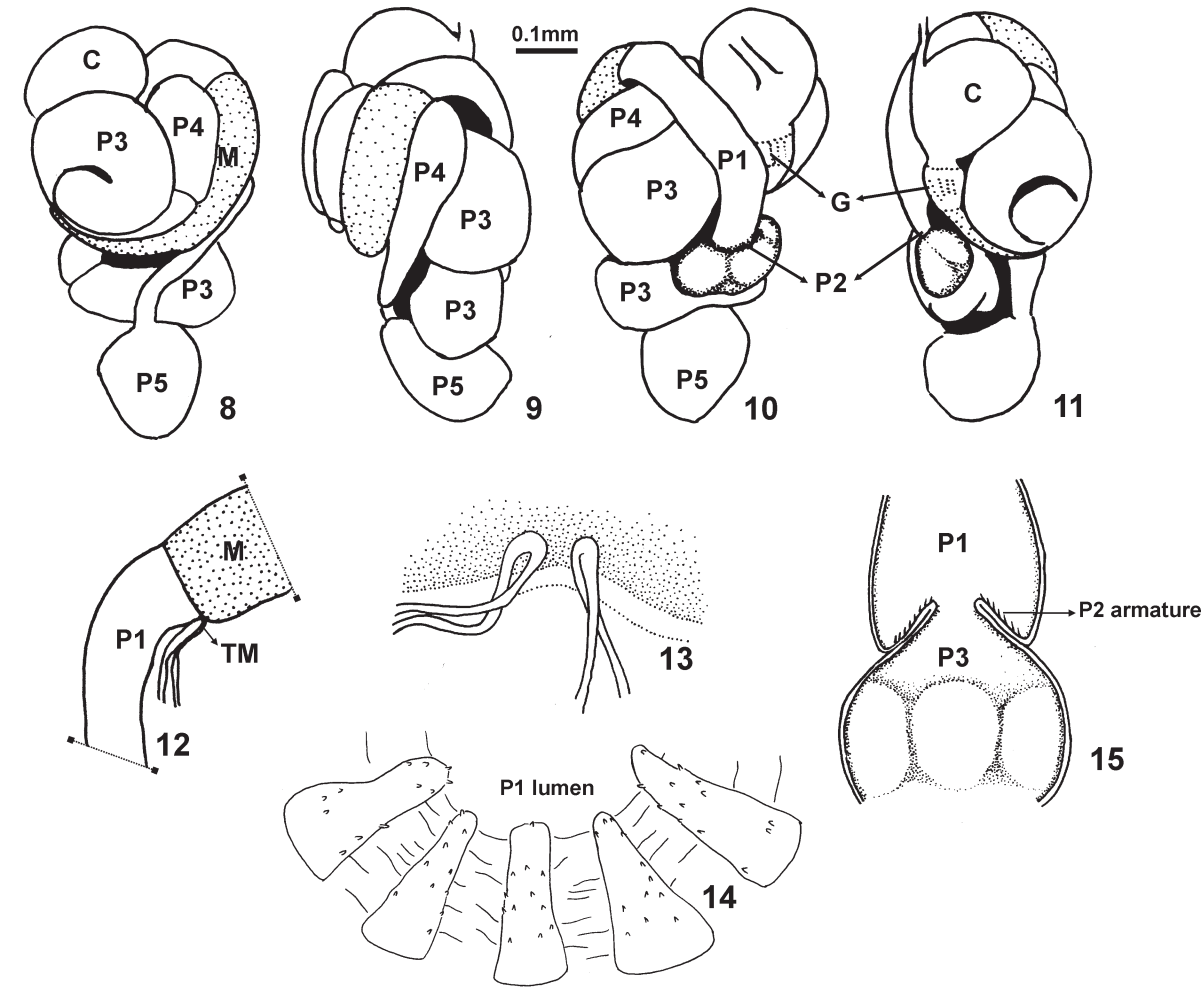
*Sinqasapatermes sachae*. **8–11** worker gut *in situ* respectively from dorsal, right, ventral and left views **12–13** Malpighian tubules attachment **14** worker enteric valve armature **15** scheme of worker enteric valve configuration. **C** crop **G** gizzard **M** mesenteron, stippled **P1** first proctodeal segment **P2** enteric valve **P3** paunch **P4** colon **P5** rectum **TM** Malpighian tubules.

*Worker* (Figs 3–15). Head capsule with numerous erect bristles and few short hairs over entire surface. Postclypeus with six bristles at anterior margin; labrum with numerous bristles. Pronotum with few short hairs on both margins; mesonotum with four bristles and metanotum with six bristles. Tergites with decumbent bristles over surface plus few erect bristles toward posterior margin. Sternites with decumbent bristles over surface plus erect ones at posterior margin. Legs with two long bristles, first proximal and second at middle on external surface of all tibiae among shorter ones. Head capsule and thoracic nota whitish; digestive tube visible through abdominal sclerites. Mandibles and digestive tube, under genus description. Measurements (mm) of seven workers are given as ranges: WH, 0.48–0.50; DA–M1+2, 0.05–0.06; LT, 0.44–0.48. Ratio: WH/LT, 1.04–1.14.

#### Etymology.

Noun in apposition taken from Quechua, *sacha*, meaning forest.

#### Remarks.

Figs 5 and 7 illustrate two sets of mandibles, which represent variation among workers of *Sinqasapatermes sachae* from the same sample. Those workers have not differentiation in coloration, pilosity, size or any other morphological characteristic to assume they belong to different instars.

## Supplementary Material

XML Treatment for Sinqasapatermes

XML Treatment for Sinqasapatermes sachae
